# Maize annexin genes *ZmANN33* and *ZmANN35* encode proteins that function in cell membrane recovery during seed germination

**DOI:** 10.1093/jxb/ery452

**Published:** 2019-01-10

**Authors:** Fei He, Canhong Gao, Genyuan Guo, Jun Liu, Yue Gao, Ronghui Pan, Yajing Guan, Jin Hu

**Affiliations:** 1 Seed Science Center, Institute of Crop Science, College of Agriculture and Biotechnology, Zhejiang University, Hangzhou, China; 2 Department of Seed Science and Industry, College of Agronomy, Anhui Agricultural University, Hefei City, China

**Keywords:** Annexins, chilling, imbibition, membrane integrity, membrane recovery, ZmANN33/35

## Abstract

Plasma membrane (PM) recovery from the impaired dry state is essential for seed germination, but its underlying mechanism remains unclear. In this study, we found that *ZmANN33* and *ZmANN35*, two annexin genes in maize, encode proteins that participate in PM recovery during seed germination. The expression of both genes was up-regulated during seed germination and strongly repressed by chilling (either 15 or 5 °C) as compared with the normal temperature (25 °C). In addition, the increased membrane damage caused by chilling imbibition was correlated with suppressed expression of *ZmANN33* and *ZmANN35*, while rapid recovery of their expression levels accompanied the rescue of the damaged membrane. Arabidopsis seedlings ectopically expressing *ZmANN33* or *ZmANN35* had longer seedling length than wild-type (WT) plants during the recovery period after 3 d of chilling stress, indicating the positive roles of these two gene products in the plant’s recovery from chilling injury. Moreover, these transgenic seedlings had lower lipid peroxidation and higher peroxidase activities than WT during the recovery period. Consistently, root cells of these transgenic seedlings had more intact PM after chilling stress, supporting the proposition that ZmANN33 and ZmANN35 contribute to the maintenance of PM integrity. The enhanced PM integrity is likely due to the accelerated exocytotic process after chilling stress. We also showed that both ZmANN33 and ZmANN35 localized in the cytosol near the plasma membrane. Thus, we conclude that *ZmANN33* and *ZmANN35* play essential roles in membrane recovery during maize seed germination.

## Teaser

Maize annexin genes *ZmANN33* and *ZmANN35* encode proteins that contribute to cell membrane recovery during maize seed germination.

## Introduction

Rapid and normal seedling establishment is essential to maize (*Zea mays* L.) production. However, as a thermophilic crop, maize is susceptible to chilling injury when sown in early spring, leading to severe inhibition of seed germination and seedling growth (Prasad, 1996). Germination is a complex process during which the seed re-activates essential cellular events from a quiescent state, including various metabolic reactions and signal transduction pathways (Nonogaki *et al.*, 2010). The germination process can be divided into three phases based on the time course of water uptake, namely (i) seed imbibition, (ii) a lag phase with no net loss or gain of water, and (iii) embryo elongation resulting in some portion of the embryo protruding from the covers (Bewley *et al.*, 2013). During the early stage of seed imbibition, the cell membrane is not yet fully repaired; a massive leakage of cellular solutes tends to occur, especially under low temperature (Bewley *et al.*, 2013). Cell membrane repair is required for seed germination and subsequent seedling establishment, but the underlying mechanisms remain unclear and are among the most interesting and important topics in seed science (Han and Yang, 2015).

With a domain with phospholipid-binding ability, plasma membrane-bound annexins on the cytosolic side in animals are able to interact with negatively charged membrane surfaces in a Ca^2+^-dependent manner (Gerke *et al.*, 2005). Lennon *et al.* (2003) reported that ANNEXINS A1 and A2 could associate with DYSFERLIN and take part in mechanically induced exocytotic patch repair of injured skeletal myotubes. McNeil *et al.* (2006) found that ANNEXIN A1 was required for repairing the plasma membrane after laser-induced injury in HeLa cells. Annexins are found to have various additional functions in animal cells, including signal transduction, free cytosolic Ca^2+^ homeostasis, exocytosis and endocytosis, membrane organization, and cytoskeletal dynamics (Gerke and Moss, 2002; Hill *et al.*, 2003; Gerke *et al.*, 2005). Among them is the direct participation of annexins in repairing animal membrane lesions, raising a reasonable inference that their counterparts in plants may have similar functions and therefore be possibly critical for seed germination.

Annexin genes have been identified across various monocot and dicot plant species. Plant annexins have only one or two conserved annexin repeats and a short N-terminal region (Hofmann *et al.*, 2000), which is structurally different from their animal counterparts. Some studies recently claimed a role for annexins in seed germination enhancement, especially under stress conditions (Yadav *et al*., 2016; Ijaz *et al*., 2017; Ahmed *et al*., 2017). NnANN1, a heat-induced annexin in sacred lotus, was found to be predominantly expressed during seed germination. Ectopic expression of *NnANN1* in Arabidopsis showed an increase in the final percentage germination under heat stress (Chu *et al.*, 2012). In maize, ZmANN33 and ZmANN35 were first identified in coleoptiles (Blackbourn *et al.*, 1991). They had been shown to form a Ca^2+^-permeable conductance in planar lipid bilayers and exhibited *in vitro* peroxidase activity, suggesting a role in signaling of reactive oxygen species (Laohavisit *et al.*, 2009; Laohavisit and Davies, 2011). Also, ZmANN33 and ZmANN35 were found to respond to physiological Ca^2+^ levels and stimulate exocytosis in root cap cells (Carroll *et al.*, 1998). So far, 12 members of the maize annexin gene family have been identified through a genome-wide analysis, and all of them exhibit distinct expression patterns regarding spatiotemporal specificities (Zhou *et al.*, 2013). These findings suggested their essential roles in regulation maize development. However, the mechanisms of how they are involved in membrane repair during seed imbibition have not yet been described.

In this study, we investigated the functions of two maize annexin proteins, ZmANN33 and ZmANN35, during seed germination. The expression of both annexin genes was up-regulated during germination but partially suppressed by chilling stress. The repair of damaged membranes during early imbibition was closely correlated with the expression levels of *ZmANN33/35*. Ectopic expression of *ZmANN33* or *ZmANN35* in Arabidopsis was conductive to the seedlings’ recovery from chilling stress to some extent and better PM integrity as well. Our results demonstrated that ZmANN33 and ZmANN35 contribute to membrane recovery during maize seed germination.

## Materials and methods

### Plant materials

The seeds of chilling-sensitive maize (*Zea mays* L.) inbred line Mo17 (Zheng *et al.*, 2006) were used in this study. Dry seeds were imbibed between wet filter papers in a growth chamber at 25 °C, and sampled at 0, 3, 6, 9, 12, 24, and 36 h after imbibition (hai) until 50% radicle protrusion. The fresh weights of samples were recorded to determine the time-course of water absorption. Several critical points during seed germination were determined as 9, 24, 32, and 54 hai. Then, dry seeds were respectively subjected to 25 and 15 °C with 12 h/12 h light–dark cycles, and sampled at 0, 9, 24, 32, and 54 hai for electrolyte leakage tests and analysis of annexin expression.

Because the membrane damage mainly occurred at the early stage of seed imbibition, seeds were imbibed in 5 °C water for 2 h and then kept at 25 °C for 7 h for recovery (5–25 °C). Sampling time points were set as 0, 0.5, 2, 3, 6, and 9 hai to further determine the membrane injury of maize embryos and expression levels of *ZmANN33* and *ZmANN35.* For the purpose of studying the effect of calcium ions on annexin expression, 5 mM EGTA (a Ca^2+^ chelator) solutions were used for contrastive analyses.

Embryos of all samples were collected rapidly, frozen in liquid nitrogen, and stored at −80 °C for subsequent analysis.

### Measurements of physiological parameters

The electrolyte leakage (EL) as an index for membrane permeability was determined based on the method described by Liu *et al.* (2000). Seeds were soaked in 50 ml of deionized water at 25 °C for 24 h, and the value EL_1_ was tested using a conductivity meter (DDS-11A, INESA, Shanghai, China). The samples were then boiled for 30 min, and cooled to 25 °C for measuring the value EL_2_. The EL value of deionized water was used as EL_0_. The relative electrolyte leakage was calculated according to the formula: EL=(EL_1_−EL_0_)/(EL_2_−EL_0_).

The malondialdehyde (MDA) concentration was measured according to Wang *et al.* (2013). About 0.1 g of plant sample was ground in 5 ml of 0.05 M sodium phosphate buffer (pH 7.8) and centrifuged at 10 000 *g* for 15 min. The supernatant was used for MDA concentration determination.

For testing catalase (CAT) and peroxidase (POD) activities, about 0.1 g of seedlings were ground in 5 ml of 0.05 M sodium phosphate buffer (pH 7.8) and centrifuged at 10 000 *g* for 15 min. Afterwards, the supernatant was used for activity analysis according to Pinhero *et al.* (1997).

Measurement of membrane ATPase activity was done using an assay toolkit (Cablebridge, Shanghai, China) to evaluate the release of inorganic phosphate (Ohnishi *et al.*, 1975).

### Gene expression analysis by quantitative RT-PCR

Total RNA of each sample was extracted and purified using the Quick Total RNA Isolation Kit (Waryong, Beijing, China). The quality and concentration of RNA were checked by the NanoDrop system (Thermo Scientific, Wilmington, DE, USA). Approximately 500 ng RNA was reverse-transcribed using PrimeScript™ RT reagent Kit (Takara, Dalian, China). Real-time PCR reactions were carried out using the CFX96TM Real-Time PCR Detection System (Bio-Rad, Hercules, CA, USA). Twenty microliters of reaction sample in each tube contained 1 μl of diluted cDNA, 0.8 μl of reverse and forward primers for each, 7.4 μl of ddH_2_O and 10 μl of the AceQ qPCR SYBR Green Master Mix (Vazyme, Nanjing, China). Primer were designed with Primer 5 software and the maize *ACTIN* gene or Arabidopsis *UBIQUITIN* was used as the control. Transcript abundance was calculated using the relative 2^−ΔΔ*C*T^analytical method. Five biological replicates were conducted, and each of them was technically repeated three times. All data were expressed as mean ±SD after normalization.

### Cloning of maize annexins

cDNAs for annexin p33 and p35 have been identified previously (Battey *et al.*, 1996). The specific primers containing the restriction sites *Nco* I and *Bst*E II were used to amplify open reading frames of *ZmANN33* (X98244.2) and *ZmANN35* (X98245.1). The PCR products were cloned into pCAMBIA1301 to generate recombinant plasmids, in which the *ZmANN33* and *ZmANN35* sequences were under the control of the strong constitutive CaMV35S promoter. The constructs were transformed into *Agrobacterium* strain EHA105 and then introduced into the genome of WT Arabidopsis plants (Columbia ecotype, Col), using the floral dip method (Clough and Bent, 1998), for transgenic studies. The transgenic lines were screened using hygromycin and PCR selection. A total of 10 (*ZmANN33*) and eight (*ZmANN35*) independent transgenic lines were obtained, and plants developed from T3 seeds of four representative individual lines, including *ZmANN33*OE-1, *ZmANN33*OE-2, *ZmANN35*OE-7, and *ZmANN35*OE-24, were selected for the subsequent analyses.

### Subcellular localization of ZmANN33 and ZmANN35

Transient expressions of ZmANN33 and ZmANN35 and their co-localization analyses were performed in tobacco leaves (Boruc *et al.*, 2010). The coding sequences of ZmANN33 and ZmANN35 were respectively cloned into the pEASY^TM^-T5 Zero cloning vector. Then, the *ZmANN33* sequence was isolated by *Sal*I/*Bam*HI double-digestion and shuttled into the pCV vector to fuse it to C-terminus of mCherry fluorescent protein, and expression vector pCV-mCherry-ZmANN33 was obtained. The pCV-GFP-ZmANN35 vector was generated using a similar strategy. These two constructs were transformed into *Agrobacterium* strain EHA105, and the resulting *Agrobacterium* cells were grown, harvested, and resuspended (OD_600_=1) in a buffer composed of 10 mM MgCl_2_, 10 mM MES (pH 5.6), and 200 μM acetosyringone for 1–2 h at room temperature before being infiltrated into fully expanded leaves of *Nicotiana benthamiana* plants. For co-localization experiment, the bacterial suspensions of *ZmANN33* and *ZmANN35* were equal-proportionally mixed and used for infiltration. Their transient transformation of tobacco leaves was performed according to Voinnet *et al.* (1998). After 3 d, the green fluorescent protein (GFP) or mCherry fluorescence was visualized under a confocal microscope (Leica TCS SP5, Germany) for determining the proteins’ subcellular localization.

### Chilling stress on the transgenic Arabidopsis seedlings

WT and T3 transgenic Arabidopsis seeds were sown on standard 1/2 MS agar medium with or without 5 mM EGTA and kept at 4 °C for 3 d before transfer to 23 °C under cycles of 16 h light and 8 h darkness. The 4-day-old seedlings were chilling treated at 1 °C for 3 d and then grown at 23 °C for an additional 2 d for recovery. These 7- (after 3 d chilling stress) and 9-day-old seedlings (after 2 d recovery) were collected for determination of their physiological parameters, including EL, MDA content, CAT, and POD activities.

### Fluorescence staining of transgenic Arabidopsis roots

PM integrity analysis was performed as previously described by Schapire *et al.* (2008). Seven-day-old WT and transgenic Arabidopsis seedlings grown under normal conditions were chilled at 1 °C for 3 h followed by immediate incubation with 50 µM FM4-64 (a lipophilic probe binding to the outer surface of the PM; Life technologies, USA) for 5 min. These roots were imaged using a confocal microscope (LSM780, Zeiss, Germany) and compared with the ones without chilling treatment (controls).

To detect the exocytosis of transgenic Arabidopsis lines, the stained seedlings were exposed to 50 µM brefeldin A (BFA; an intracellular protein transport inhibitor) (Beyotime, Shanghai, China) for 1.5 h (Deng *et al.*, 2015). Intracellular BFA bodies aggregated and expanded gradually due to the inhibitory effect of BFA on exocytosis. BFA was then washed out in liquid 1/2 MS medium for 1.5 h. Fluorescence of FM4-64 uptake was visualized with a confocal microscope (488 nm excitation and 620–710 nm emission) before and after BFA washing and quantified as the ratio of fluorescence intensity of intracellular bodies to that of the whole cell.

### Statistical analysis

All experiments were repeated at least three times and the data were presented as the mean ±standard error (SE). The data were subjected to analysis of variance (ANOVA) to detect statistical differences using the SAS statistics program, and the means were compared by the least significant difference (LSD) test at the 0.05 level of significance.

## Results

### Chilling injury during maize seed germination

**Fig. 1.**
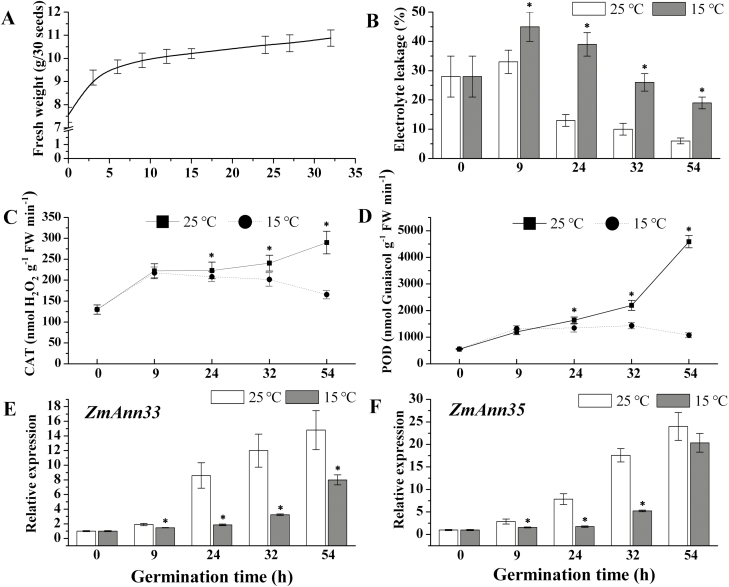
The expression analysis of *ZmANN33* and *ZmANN35* during maize germination. (A) Time course of water absorption in germinating maize seed. The fresh weights of germinating seeds on water were recorded at different time points. (B) Electrolyte leakage assay of germinating maize seeds under normal (25 °C) or chilling condition (5 °C). The data are presented as the mean ±standard deviation (SD). (C, D) Two typical antioxidant enzymes activities (catalase and peroxidase) in maize embryos during normal or chilling germination. (E, F) Quantitative RT-PCR assay for expression analysis of *ZmANN33* (E) and *ZmANN35* (F) during normal or chilling germination. The relative mRNA levels were normalized with reference gene (actin) and based on dry seed as reference sample set to 1.0. *Significant difference at α=0.05, LSD test.

The water absorption curve of maize seeds could be divided into three phases (Fig. 1A). Initially, seed imbibition with rapid water absorption occurred from 0 to 9 h, which was followed by a lag phase of water absorption until radicle protrusion from the seed cover at about 32 hai. During the post-germination stage, the radicle grew rapidly to the length of the seed at 54 hai. We selected 0 (dry seeds), 9, 24, 32, and 54 hai as sampling time points during seed germination and seedling establishment.

The highest value of electrolyte leakage (EL) was detected at 9 hai and then decreased under both normal and chilling conditions (Fig. 1B). However, the EL of germinating seeds under chilling condition was significantly higher than that obtained under normal condition. This observation indicates that although the cellular membrane system was in an impaired state during early seed imbibition, the impairment was more severe under chilling conditions. Besides, the activities of CAT and POD decreased considerably during chilling imbibition compared with the normal condition (Fig. 1C, D), which may reduce the seeds’ capability to repair membrane damage.

### Low temperature reduced the expression of *ZmANN33* and *ZmANN35* during germination

The transcript levels of *ZmANN33* and *ZmANN35* gradually increased during seed germination and reached the highest values at 54 hai under 25 °C. However, although these two genes’ expression was up-regulated also when the seeds were germinated at 15 °C, the increase was not as great as that at 25 °C (Fig. 1E, F). It is worth noting that there was a decrease in seed electrolyte leakage from 9 to 54 hai (Fig. 1B), which was coincident with the increased expression of annexins, suggesting the involvement of ZmANN33 and ZmANN35 in maintaining the membrane integrity during germination.

### Annexin expression and membrane injury during early imbibition of maize seeds

**Fig. 2.**
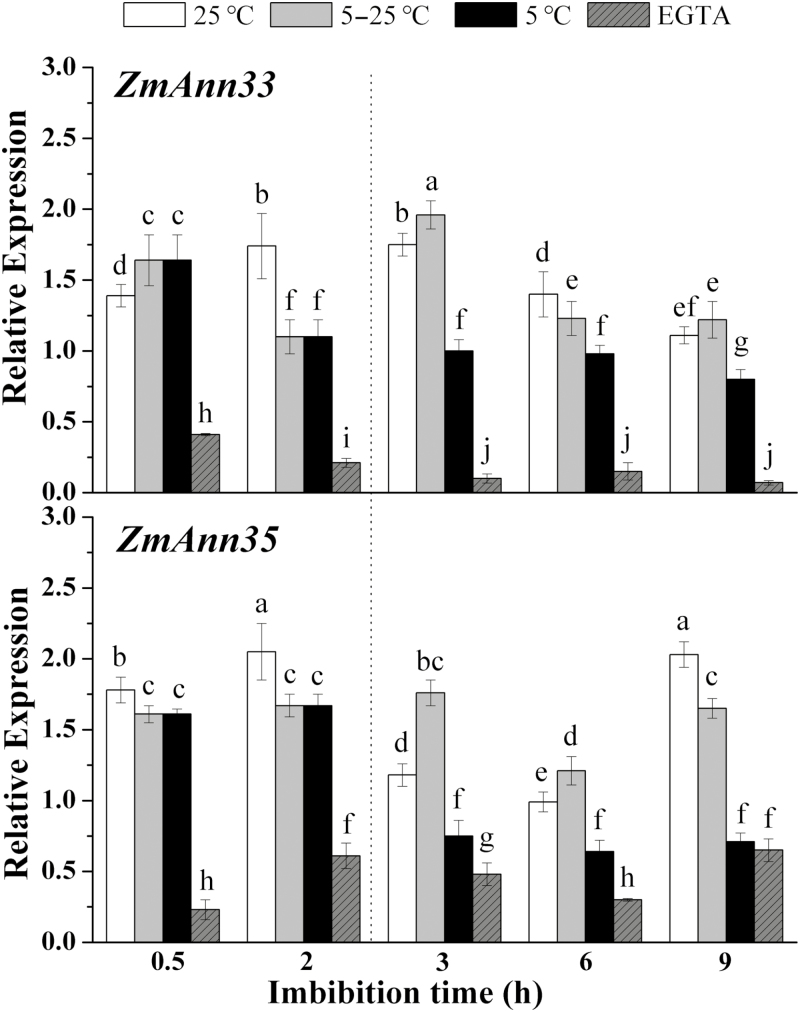
The expression profiles of *ZmANN33* and *ZmANN35* during early imbibition of maize seed. Transcript levels of *ZmANN33* and *ZmANN35* during imbibition were evaluated under different treatments. ‘25 °C’ columns: seeds were imbibed in water at 25 °C; ‘5–25 °C’ columns: seeds were soaked in water for 2 h at 5 °C, and then transferred to 25 °C for recovery for 7 h; ‘5 °C’ columns: seeds were imbibed in water at 5 °C for 9 h. Additionally, exogenous EGTA was added to investigate the effects of Ca^2+^ on *ZmANN33* and *ZmANN35* expression. ‘EGTA’ columns: seeds were imbibed in 5 mM EGTA solution at 25 °C. Embryos under each treatment were sampled at 0, 0.5, 2, 3, 6, and 9 h after imbibition. The relative mRNA levels were normalized to the reference gene (actin) and based on dry seed as reference sample set to 1.0. Different letters indicate a significant difference at α=0.05, LSD test.

The repair of injured membrane was proposed to be activated during early seed germination (Weitbrecht *et al.*, 2011). Thus, we investigated the potential role of annexins during the early imbibition stage under chilling condition. Transcript levels of *ZmANN33*/*35* increased immediately upon the initiation of water uptake and remained relatively high (about 2-fold) compared with dry seeds during the first several hours of imbibition under 25 °C (Fig. 2). To obtain a transient chilling injury, seeds were imbibed at 5 °C for 2 h, followed by recovery at 25 °C (‘5–25 °C’ columns, Fig. 2). As a comparison, seeds were imbibed under 5 °C without a recovery period (‘5 °C’ columns, Fig. 2). Under continuous 5 °C imbibition (‘5 °C’ columns, Fig. 2), the expression of *ZmANN33/35* was gradually down-regulated. At 3 hai, the expression of *ZmANN33/35* after 1 h recovery (‘5–25 °C’ columns, Fig. 2) was dramatically higher than that without recovery (‘5 °C’ columns, Fig. 2). In addition, the expression of both *ZmANN33* and *ZmANN35* was significantly suppressed in the presence of the Ca^2+^ chelator EGTA under 25 °C (‘EGTA’ columns, Fig. 2).

**Fig. 3.**
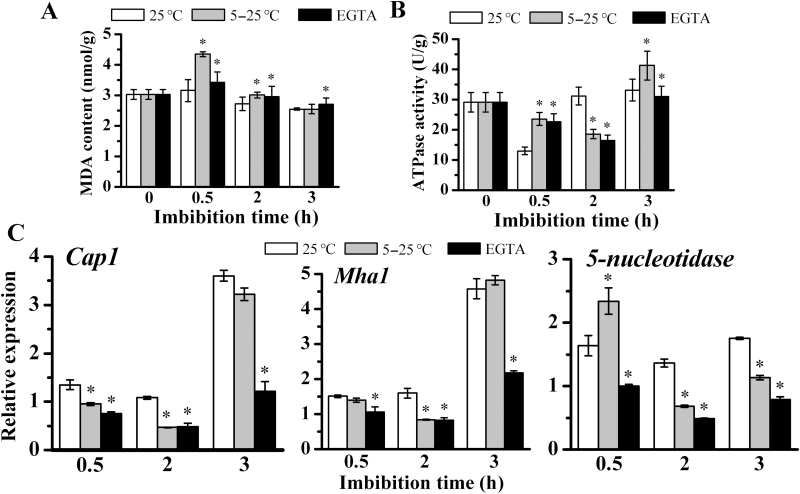
Membrane injury of maize embryos during early imbibition. (A, B) Extent of cell membrane injury during maize early imbibition was evaluated by the measurement of MDA content (A) and PM ATPase activity (B). Embryos were collected at 0.5, 2, and 3 h after imbibition and from dry seeds. For descriptions of each treatment, see Fig. 2. (C) Transcripts levels of three typical membrane enzymes were examined. Maize actin gene was used as an internal control. The results are expressed as relative values based on dry seed as reference sample set to 1.0. *Significant difference in the same sampling time from different treatments at α=0.05, LSD test.

Moreover, two membrane-related physiological indicators, MDA content (Kuk *et al.*, 2003) and ATPase activity (Liu and Dong, 2011), were investigated to indicate the degree of membrane injury. The MDA content showed a transient increase at 0.5 hai, followed by a gradual decrease (Fig. 3A). At 0.5 hai, the MDA level in embryos at 5 °C was significantly higher than that at 25 °C, implying more severe damage to cell membranes under chilling stress. The presence of EGTA led to higher MDA contents as compared with continuous 25 °C treatment during 3 h of imbibition (Fig. 3A). The ATPase activity was significantly enhanced at 0.5 hai after chilling and EGTA compared with the 25 °C treatment (Fig. 3B), which might be due to the protective responses of the plant. EGTA and chilling (5 °C) considerably suppressed ATPase activity, which, however, increased again after 1 h of recovery (Fig. 3B).

Besides, the expression of the calcium ATPase gene (*CAP1*) significantly decreased during 2 h chilling imbibition, and after 1 h recovery rapidly increased to the level of 25 °C. EGTA inhibited *CAP1* expression during 3 h of imbibition. Another membrane ATPase gene, H^+^-ATPase 1 (*MHA1*), showed a similar expression pattern to *CAP1.* 5′-Nucleotidase is a membrane-anchored protein that is related to membrane stability under chilling stress (Jian *et al*., 1982). Its expression increased significantly and was higher than that at 25 °C at 0.5 hai. From 0.5 to 2 h, its expression declined rapidly, but 1 h recovery was enough for it to increase. EGTA had negative effects on its expression during early seed imbibition.

As above, we concluded that chilling caused severe damage to the membrane system of imbibed seeds and that the resultant damage may be alleviated by recovery under normal temperature.

### Subcellular localization of ZmANN33 and ZmANN35

**Fig. 4.**
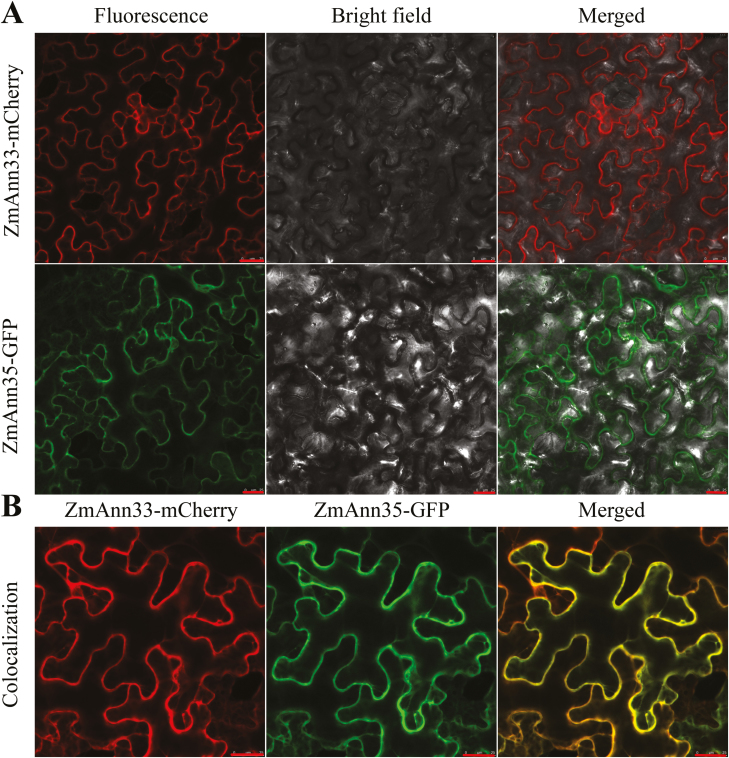
Subcellular localization of ZmANN33 and ZmANN35. (A) Transient expression of *ZmANN33* and *ZmANN35* in tobacco leaf cells. Full length cDNA of *ZmANN33* and *ZmANN35* were appended with mCherry and GFP, respectively. (B) Co-localization of two annexin genes. Bar=25 µm.

To further define the possible subcellular localization of ZmANN33 and ZmANN35, the ZmANN33–mCherry and ZmANN35–GFP constructs were generated and transiently expressed in tobacco mesophyll cells to examine their subcellular localization. Confocal microscopy revealed that both proteins were mostly located in the cytosol near the plasma membrane (cell periphery) with a similar pattern (Fig. 4A). Further experiments confirmed their co-localization near the PM (Fig. 4B). Hence, ZmANN33 and ZmANN35 proteins were possibly located near the PM.

### Ectopic expression of ZmANN33 or ZmANN35 promoted seedling recovery from chilling stress

To further investigate the function of maize annexins, ZmANN33 and ZmANN35 were ectopically expressed in Arabidopsis plants. Four representative independent lines, *ZmANN33*OE-1, *ZmANN33*OE-2, *ZmANN35*OE-7, and *ZmANN35*OE-24, were used in this study. The RT-PCR analysis revealed that the transgenes’ expression levels in the above plants were 1.7-, 2.5-, 1.2-, and 2.4-fold, respectively, of the average value of analysed transgenic lines (Supplementary Fig. S1 at *JXB* online). The 4-day-old seedlings of transgenic lines and WT were subjected to 1 °C chilling for 3 d to determine the effects of chilling stress on growth. In addition, exogenous EGTA was added to the growth medium to investigate the plant’s responses to Ca^2+^ deficit.

After 3 d of chilling stress on normal medium, there were no noticeable differences in seedling morphology, root length, and EL among the transgenic lines and WT (Supplementary Fig. S2). However, transgenic lines had shorter root length and lower EL than WT did on EGTA-containing medium (Supplementary Fig. S2B, C), suggesting the importance of Ca^2+^ for annexins function.

**Fig. 5.**
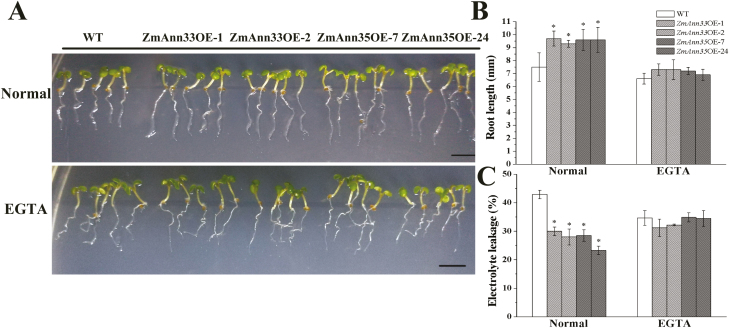
Ectopic expression of *ZmANN33/35* promoted seedling recovery from chilling stress. (A) Phenotypes of Arabidopsis seedlings after 2 d of recovery from chilling stress (1 °C). *ZmANN33* ectopic expression Arabidopsis seedlings (ZmANN33OE-1, ZmANN33OE-2), *ZmANN35* ectopic expression seedlings (ZmANN35OE-7, ZmANN35OE-24) and WT (Col) were grown for 4 d at 23 °C on standard 1/2 MS medium and 1/2 MS medium supplemented with 5 mM EGTA. Then they were subjected to 1 °C for 3 d. After the chilling treatment, two kinds of transgenic seedlings and WT under different treatments were transferred to 23 °C for 2 d recovery. Bar=5 mm. (B, C) Root length and electrolyte leakage of different Arabidopsis seedlings corresponding to (A). *Significant difference from WT at α=0.05, LSD test. (This figure is available in color at *JXB* online.)

During the recovery under 23 °C, the growth of transgenic seedlings was promoted on the normal medium (Fig. 5A). Their root length and EL were significantly higher and lower than WT, respectively (Fig. 5B, C), suggesting better PM integrity during the recovery period in *ZmANN33/35* transgenic seedlings. Seedlings grown on the medium with EGTA, however, showed no evident differences between transgenic lines and WT (Fig. 5).

### Ectopic expression of ZmANN33 or ZmANN35 enhanced antioxidant capability of Arabidopsis seedlings

**Fig. 6.**
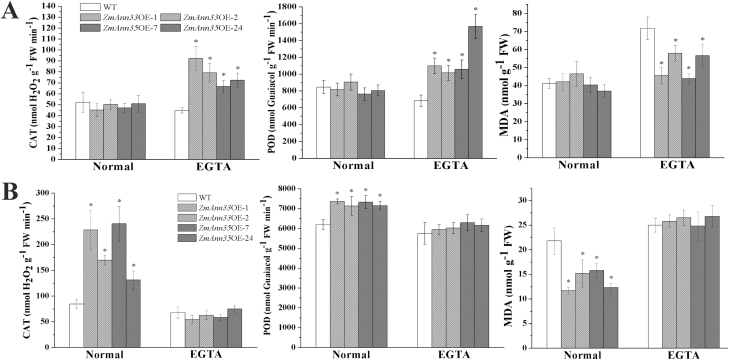
The antioxidant enzyme activities and MDA contents in transgenic seedlings. The typical antioxidant enzymes activities (CAT, SOD) and MDA contents were examined. (A) Physiological activity of Arabidopsis seedlings under different treatments after 3 d chilling stress. (B) Physiological activity of Arabidopsis seedlings under different treatments after 2 d recovery period from chilling stress. *Significant difference from WT at α=0.05, LSD test.

CAT and POD are major antioxidant enzymes involved in modulation of intracellular ROS levels. On normal medium, CAT and POD activities and MDA content in four transgenic lines showed no difference when compared with WT during 3 d of chilling stress (Fig. 6A). However, during the recovery period, transgenic lines had notably enhanced activity of these two enzymes and decreased MDA content as compared with WT seedlings (Fig. 6B).

Under chilling stress, *ZmANN33* and *ZmANN35* transgenic seedlings on the EGTA medium exhibited higher enzyme activities and lower MDA content compared with WT. During the recovery period, the presence of EGTA made no significant difference between transgenic and WT plants.

### Ectopic expression of ZmANN33 or ZmANN35 help to maintain PM integrity

**Fig. 7.**
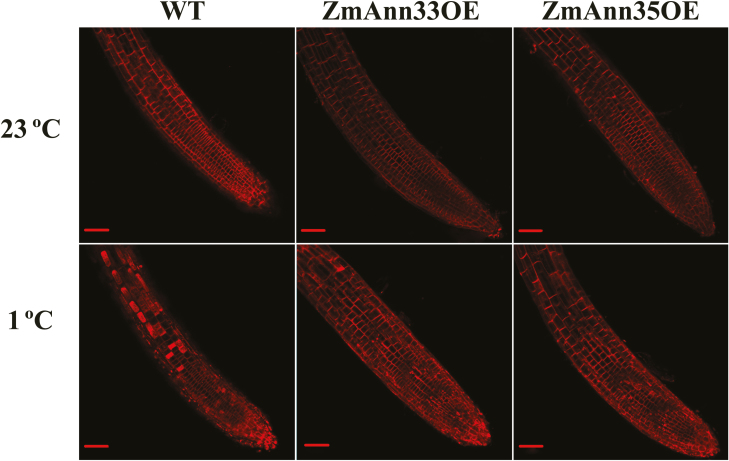
Enhanced plasma membrane integrity in transgenic seedling cells under transient chilling stress. Two kinds of transgenic lines and WT seeds were germinated on 1/2 MS medium for 7 d at 23 °C. Then they were placed under 1 °C for 3 h to obtain a transient chilling stress, followed by FM4-64 (50 µm) staining for 5 min (‘1 °C’ row). Staining of cytoplasm is the result of loss of plasma membrane integrity. Control seedlings were treated at 23 °C for 3 h (‘23 °C’ row). Representative roots are shown; bar=50 µm. For each test, at least five biological replicates were examined.

With injured PM, the intracellular membranes can be easily stained by an influx of the fluorescent dye FM4-64 (Dhonukshe *et al.*, 2006; Schapire *et al.*, 2008). Under 23 °C, root cells of both transgenic seedlings and WT showed clearly intact outlines (Fig. 7, ‘23 °C’ row), indicating the accumulation of FM4-64 outside of cells. After transient chilling stress (Fig. 7, ‘1 °C’ row), most cells in the root elongation zone and root tip of WT seedlings were strongly stained with FM4-64, indicating disruption of their PM. However, the numbers of cells with damaged PM were remarkably smaller in *ZmANN33/35* transgenic plants than those in WT, suggesting that ectopic expression of ZmANN33 or ZmANN35 can help to maintain PM integrity during chilling stress.

### Ectopic expression of ZmANN33 or ZmANN35 enhanced exocytosis in Arabidopsis roots

**Fig. 8.**
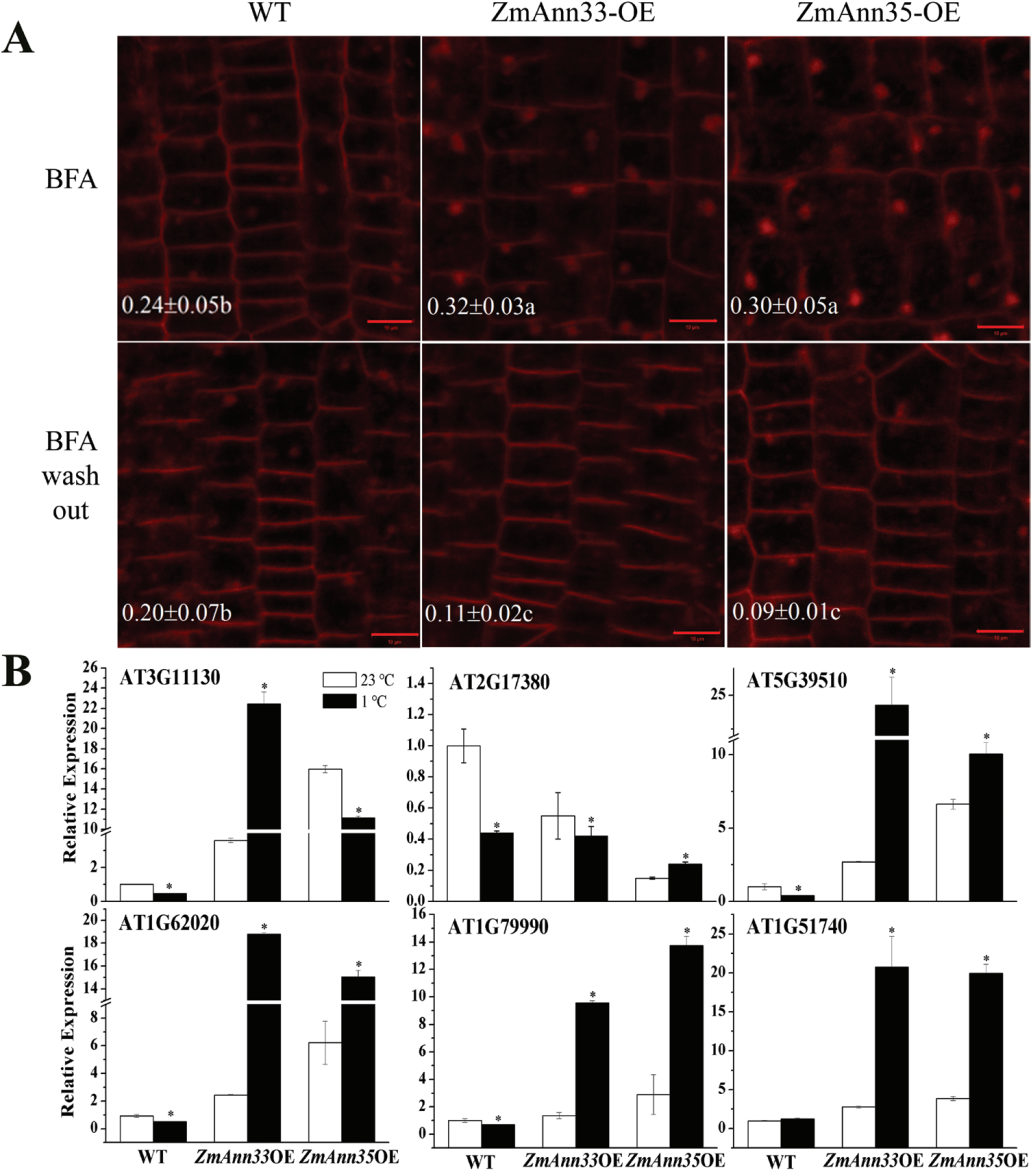
Ectopic expression of *ZmANN33/35* enhanced exocytosis in Arabidopsis roots. (A) Observation of exocytosis activity in different Arabidopsis roots. ZmANN33-OE, ZmANN35-OE and WT seeds were germinated on 1/2 MS medium for 7 d at 23 °C. Then they were placed at 1 °C for 3 h to obtain a transient chilling stress, immediately followed by FM4-64 (50 µm) staining for 5 min. After staining, seedlings were exposed to 50 µM Brefeldin A (BFA) for 1.5 h. Then, BFA was washed out in liquid 1/2 MS medium for 1.5 h. Fluorescence of FM4-64 uptake was observed before and after BFA washing and was quantified as the ratio of intracellular bodies’ fluorescence intensity to the whole cell. Values ±SD labeled with letters show significant differences at *P*<0.05 between seedlings and BFA treatments. Bar=10 µm. (B) Expression of intracellular vesicle transport-related genes of different Arabidopsis seedlings after chilling stress. Seven-day-old seedlings were subjected to 1 °C for 3 h and then their gene expression was analysed, including *CHC1* (clathrin heavy chain 1), *AAP19-1* (AP-1 complex subunit sigma-1), *VTI11* (vesicle transport v-SNARE 11), *AT1G62020* (coatomer subunit alpha-1), *AT1G79990* (coatomer subunit beta-1), and *SYP81* (syntaxin-81). Control treatment was at 1 °C for 3 h. The relative mRNA levels were normalized to reference gene (*UBQ10*) and based on WT at 23 °C as reference sample set to 1.0. *Significant difference from WT at α=0.05, LSD test. For each test, five biological replicates were examined.

To find out the possible mechanism of enhanced membrane integrity, we analysed the exocytotic process in transgenic Arabidopsis cells. Because BFA inhibited exocytosis but not endocytosis, BFA bodies gradually aggregated within cells. The *ZmANN33*/*35* transgenic lines exhibited bigger BFA bodies compared with WT after 3 h of chilling stress (Fig. 8A). The BFA bodies in transgenic lines were significantly reduced after BFA was washed out, while those in WT remained a comparable size to BFA-treated cells (Fig. 8A), indicating more vigorous exocytosis activity in *ZmANN33*- or *ZmANN35-*expressing seedlings than WT under chilling stress. In addition, the expression of six genes that participate in intracellular vesicle transport was almost all remarkably up-regulated in *ZmANN33* or *ZmANN35* transgenic seedlings after chilling treatment, while in WT, their expression was nearly all suppressed (Fig. 8B). These genes encode proteins that include SNAREs, SYNTAXIN, CLATHRIN, COATOMER, and members of adaptor complexes, which is consistent with the vesicle transport-related genes found in the imbibed maize embryo at the recovery stage from chilling stress (He *et al.*, 2017). These results suggest that ectopic expression of *ZmANN33* or *ZmANN35* in Arabidopsis enhances the intracellular exocytotic process.

## Discussion

Seed germination is a very complex process, in which physiological metabolism changes dramatically in a short period of time. In this study, the high value of electrolyte leakage (Fig. 1B) indicated impairment of the cell membrane systems during early imbibition of seeds, and the decrease of this value indicated subsequent repair of the damaged membrane as seed germination progressed. Given the essential function of annexins in PM resealing in animal cells (Andrews *et al.*, 2014; Draeger *et al.*, 2014), the plant annexins were proposed as possible components involved in the PM repair (Schapire *et al*., 2008; Draeger *et al.*, 2011) as cytosolic, peripheral, or integral membrane proteins (Talukdar *et al.*, 2009). However, how the repair of the damaged membrane is achieved remains unclear, and it is uncertain whether annexins benefit membrane recovery during maize germination.

The maize annexins, ZmANN33 and ZmANN35, shared 80% amino acid identity (Battey *et al.*, 1996), but their functions remained to be determined. Dramatic changes in *ZmANN33*/35 transcript levels happened during maize seed germination. Similarly, *ANNEXIN* transcript levels significantly increased during seed germination in rice (Yang *et al.*, 2007) and sacred lotus (Chu *et al.*, 2012). Besides, overexpression of annexin genes *AtANN1* and *AtANN4* in Arabidopsis (Huh *et al.*, 2010) and *SpANN2* in tomato (Ijaz *et al*., 2017) enhanced drought and salt tolerance during seed germination. Some other transgenic analyses suggested that germination behaviors of transgenic seeds were similar to WT seeds under normal condition, but under stress condition they were superior (Lee *et al.*, 2004, Chu *et al.*, 2012; Zhang *et al.*, 2015; Yan *et al*., 2016; Ahmed *et al*., 2017). Those findings implied that annexins were likely germination-related proteins and played important roles in seed germination especially under adverse conditions.

Membrane injury was easily caused by chilling, mainly due to lipid peroxidation as reported previously (Bochicchio *et al.*, 1991). Chilling imbibition significantly decreased the expression of *ZmANN33/35*, as shown in this study (Fig. 2), and accompanied aggravated membrane injury, as indicated by the increased MDA content (Fig. 3A) and the suppressed PM ATPase activities (Fig. 3B, C). During the recovery period, the membrane damage was repaired, coincident with the significant increases in *ZmANN33/35* transcript levels. Annexins are Ca^2+^-binding proteins. The presence of the extracellularly situated, membrane-impermeant Ca^2+^ chelator EGTA remarkably suppressed *ZmANN33* and *ZmANN35* transcript levels and aggravated the membrane injury in maize embryos undergoing chilling imbibition. These observations suggested that the extent of cellular membrane damage may be indicated to a certain degree by the expression levels of *ZmANN33*/*35*.

The expression of different annexin genes during germination and chilling stress can be very diverse. Unlike the decreased expression of *ZmANN33/35* during maize seed chilling imbibition, *ANNEXINs* from other species were activated by low temperature. One study in wheat found that protein levels of two wheat ANNEXINs gradually increased after cold treatment (Breton *et al.*, 2000). A proteomic analysis in poplar indicated *ANNEXIN*s were activated after cold acclimation (Renaut *et al.*, 2004). A similar result was also observed in a transcriptomic analysis of wheat, in which they found an ANNEXIN, highly similar to ZmANN33, accumulated preferentially in the leaves of two winter cultivars (Winfield *et al*., 2010). These observations were opposite to our results. One possible reason is that those studies analysed older seedlings with a relatively stable physiological state, while in our research, germinating seeds with dramatically changing physiological metabolism were used. Therefore, *ZmANN33/35* expression was likely regulated by the dynamic balance between cold response and germination. Interestingly, ZmANN33 and ZmANN35 showed highest sequence similarity with AtANN2 (Clark *et al.*, 2001) among Arabidopsis ANNEXINs. *AtANN2* expression was down-regulated in response to 4 °C cold treatment while most other annexin members in Arabidopsis were up-regulated (Cantero *et al.*, 2006). These observations suggest the diverse functions of plant ANNEXINs in response to cold stress.

There was no obvious growth advantage in *ZmANN*33/35-expressing Arabidopsis seedlings under the chilling condition in this study. However, after 2 d of recovery, transgenic seedlings showed stronger roots and less cellular membrane damage (Fig. 5), lower lipid peroxidation and higher peroxidase activities than did WT (Fig. 6). Similar results were obtained in Arabidopsis. *AtANN1* overexpressing seedlings accumulated less hydrogen peroxide and were more drought-tolerant than WT (Konopka-Postupolska *et al.*, 2009). In tobacco, ectopic expression of *BjANN1* reduced lipid peroxidation under drought, salinity, and heavy metal stresses (Jami *et al.*, 2008). The ANNEXINs were reported to possess peroxidase activity, which relied on the conserved His residue in the first annexin repeat (Gidrol *et al.*, 1996; Laohavist *et al*., 2009). Therefore, the important roles of ZmANN33 and ZmANN35 in repairing membrane damage might be to do with their possible roles in lipid peroxides and ROS regulation.

The calcium ion plays an important role in plant stress responses. Responses to cold stress in plants typically involve Ca^2+^ signaling (Chinnusamy *et al*., 2007). As distinct Ca^2+^-binding proteins, ANNEXINs are supposed to be involved in Ca^2+^ signaling in adaptive responses (Laohavisit and Davies, 2011). EGTA repressed root growth of transgenic lines under chilling stress (Supplementary Fig. S2) and eliminated the positive effect of *ZmANN33/35* ectopic expression on seedling growth during the recovery period (Fig. 5). It seems that *ZmANN33/35* ectopic expression made plants more sensitive to Ca^2+^ deficiency under low temperature. The possible reason was that as Ca^2+^-binding proteins and unconventional calcium-permeable channels, plant ANNEXINs have been implicated in Ca^2+^ signal transduction and amplification (White *et al.*, 2002; Mortimer *et al.*, 2008; Winfield *et al.*, 2010; Davies, 2014). Laohavisit *et al.* (2009) reported that maize annexins were able to modulate [Ca^2+^]_cyt_ and create a Ca^2+^ influx pathway, especially during stress responses. Several studies proposed ANNEXINs were mainly involved in stress signaling and transduction pathways in response to cold stress (Breton *et al.*, 2000; Renaut *et al.*, 2004; Winfield *et al.*, 2010). Moreover, a recent study in rice indicated that the knockout of *OsANN3* significantly decreased plant tolerance to cold stress (Shen *et al.*, 2017). Thus, ZmANNs are supposed to be involved in Ca^2+^ signaling processes in response to chilling stress. Interestingly, during chilling stress, EGTA improved the antioxidant activities and decreased MDA content in transgenic lines compared with WT (Fig. 6A). This might be caused by the protective response of transgenic plants to dual adverse effects of EGTA and chilling, and needs to be further investigated in the future.

Moreover, the fluorescence staining indicated more intact PM in the roots of transgenic plants than WT after cold treatment (Fig. 7). Hence, maize ANNEXINs were hypothesized to be membrane recovery-related proteins that function in maintaining PM integrity. Previous studies demonstrated that Ca^2+^-triggered exocytosis was essential in PM resealing (McNeil and Kirchhausen, 2005) and that ZmANN33/35 can stimulate exocytosis in root cap protoplasts by escalating the membrane capacitance (Carroll *et al.*, 1998). In our study, significantly reduced BFA in transgenic Arabidopsis root cells after the removal of BFA suggested enhanced intracellular exocytosis (Fig. 8A), which was further supported by the expression profiles of genes encoding intracellular vesicle transport-related proteins (Fig. 8B). These genes were found to be differentially expressed at the recovery stage of chilling imbibed maize seeds through transcriptome analysis and mainly encode proteins that include SNAREs, SYNTAXIN, CLATHRIN, COATOMER and members of the adaptor complexes, some of which are implicated in facilitating the intracellular vesicular trafficking in the secretory and endocytosis pathways (He *et al.*, 2017). Similarly, the expression levels of those genes from either *ZmANN33* or *ZmANN35* transgenic seedlings were almost all up-regulated after chilling treatment relative to WT. Besides, annexins were also reported to be involved in endocytosis (Futter and White, 2007), which internalized surface membrane and recycled components back to the PM. Therefore, the crucial roles of ZmANN33/35 in membrane repair or maintenance of PM integrity might be their involvement in intracellular vesicular trafficking processes.

Taken together, ectopic expression of *ZmANN33*/*35* in Arabidopsis promoted the seedlings’ recovery from chilling stress and enhanced the PM integrity. Our combined data demonstrate that the protective roles of ZmANN33/35 can be attributed to their functions in ROS elimination and exocytosis. We confirmed that *ZmANN33* and *ZmANN35* encode membrane recovery-related proteins with similar functions during maize seed germination. However, there remain many unanswered questions about the mechanism of cell membrane recovery or repair during seed germination. For example, which components do annexins interact with during membrane recovery? Are they applicable to the universal PM injury repair in plant cells, just like the Synaptotagmin1 (SYT1) in Arabidopsis (Schapire *et al.*, 2008; Yamazaki *et al.*, 2008)? How are ZmANN33/35 involved in the cold-responsive pathway? As chilling-suppressed genes, *ZmANN33/35*’s involvement in the cold-responsive pathways also remains to be further investigated. After all, the results in this study help in understanding the molecular mechanisms of seed germination and the functions of plant annexins.

## Supplementary data

Supplementary data are available at *JXB* online.

Fig. S1. The expression levels of *ZmANN33* or *ZmANN35* in the transgenic Arabidopsis plants.

Fig. S2. Growth of *ZmANN33* and *ZmANN33* transgenic Arabidopsis seedlings during chilling stress.

Supplementary Figures S1 and S2
