# Supplementary material for: Maize annexin genes ZmANN33 and ZmANN35 encode proteins that function in cell membrane recovery during seed germination
Source: J Exp Bot. 2019 Jan 10;70(4):1183–95. doi: 10.1093/jxb/ery452 (PMC6382337; doi:10.1093/jxb/ery452)
Supplement: Supplementary Figures S1 and S2 [file ery452_suppl_supplementary_figures_s1-s2.pdf]

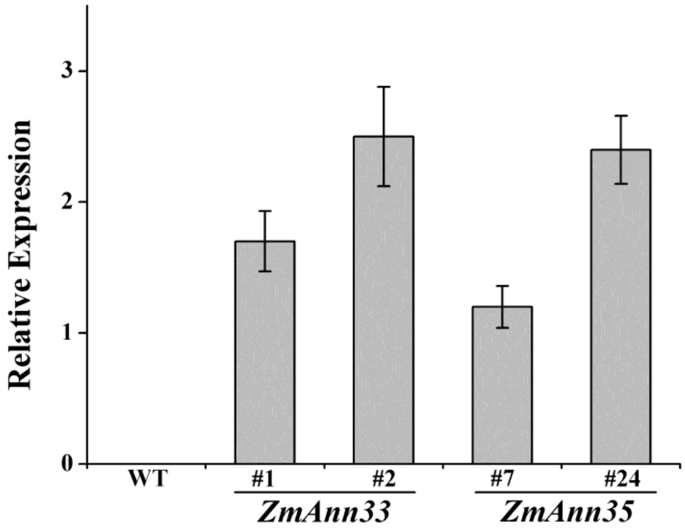

Fig. S1. The expression levels of *ZmANN33 or ZmANN35* in the transgenic *Arabidopsis* plants.

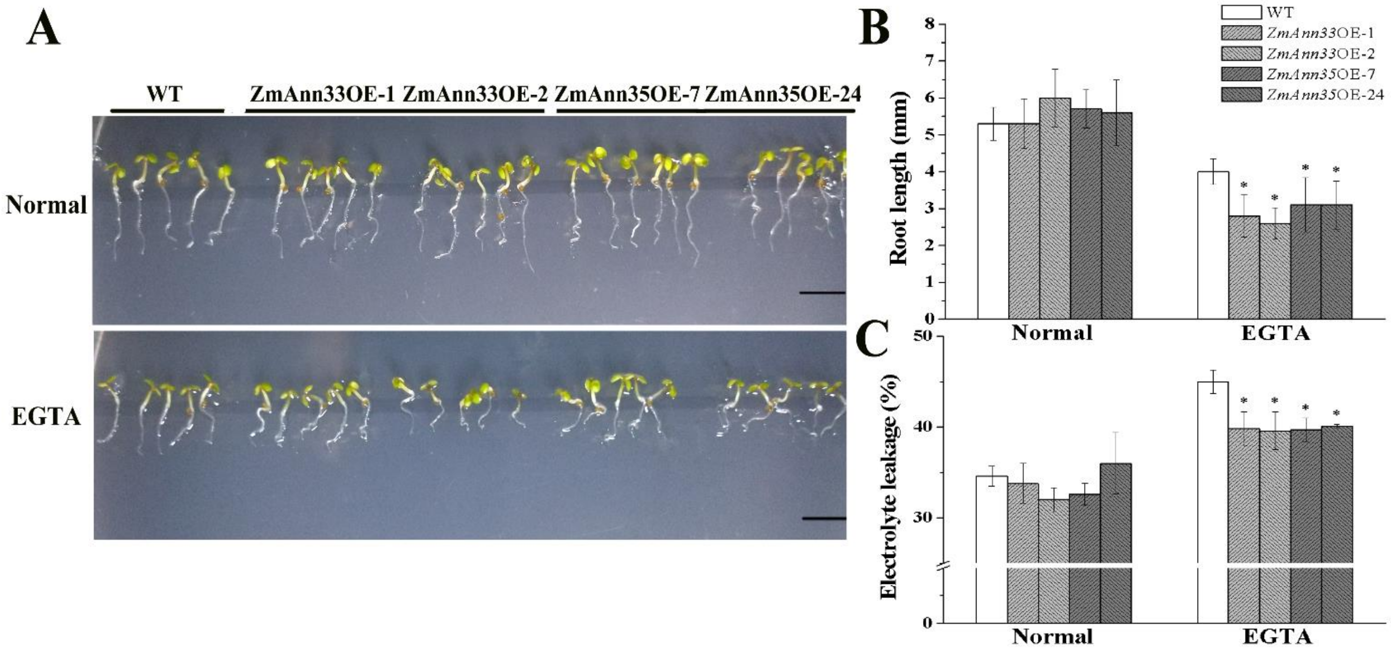

Fig. S2. Growth of *ZmANN33* and *ZmANN33* transgenic *Arabidopsis* seedlings during chilling stress.

A Phenotypes of *Arabidopsis* seedlings in response to 3 days chilling stress (1ºC). *ZmANN33* ectopic expression *Arabidopsis* seedlings (ZmANN33OE-1, ZmANN33OE-2), *ZmANN35* ectopic expression seedlings (ZmANN35OE-7, ZmANN35OE-24) and WT (Col) were grown for 4 days under 23ºC on standard 1/2 MS medium and 1/2 MS medium supplemented with 5mM EGTA. Then they were subjected to 1ºC for 3d, bar = 5mm. **B** and **C** Root length and electrolyte leakage of different *Arabidopsis* seedlings corresponding to chilling stress. * indicated a significant difference from that of WT at $\alpha = 0.05$, LSD
